# Supplementary material for: Nationwide germline whole genome sequencing of 198 consecutive pediatric cancer patients reveals a high incidence of cancer prone syndromes
Source: PLoS Genet. 2020 Dec 17;16(12):e1009231. doi: 10.1371/journal.pgen.1009231 (PMC7787686; doi:10.1371/journal.pgen.1009231)
Supplement: S1 Text — (DOCX) [file pgen.1009231.s002.docx]

**Supplementary material**

Checklist – patient phenotype

General:

Weight (kg):______________________________________________________________________________________

Height (cm):______________________________________________________________________________________

Head circumference (cm):___________________________________________________________________________

Hemihypertrophy □Yes □No _______________________________

Congenital malformations □Yes □No _______________________________

Developmental delay □Yes □No _______________________________

Head and neck:

Neck abnormalities (long/short, webbed neck):__________________________________________________________

Dysmorphic facial features (coarse facial features/prominent middle face or nose/abnormal cranial shape/retrognathia): ________________________________________________________________________________________________

Struma □Yes □No _______________________________

Other □Yes □No _______________________________

Eyes:

Telangiectasia □Yes □No _______________________________

Micro-ophthalmic eyes □Yes □No _______________________________

Hypertelorism □Yes □No _______________________________

Hypotelorism □Yes □No _______________________________

Leukocoria □Yes □No _______________________________

Strabismus □Yes □No _______________________________

Aniridia □Yes □No _______________________________

Epicanthus □Yes □No _______________________________

Other □Yes □No _______________________________

Ears:

Deafness □Yes □No _______________________________

Abnormal ear shape □Yes □No _______________________________

Low set ear □Yes □No _______________________________

Other □Yes □No _______________________________

Mouth:

Macroglossia □Yes □No _______________________________

Mucocutaneous lesions □Yes □No _______________________________

Cobblestone papules in gingiva □Yes □No _______________________________

Leukoplakia □Yes □No _______________________________

Cleft lip □Yes □No _______________________________

Cleft palate □Yes □No _______________________________

Tooth status □Good □Bad _______________________________

Other □Yes □No _______________________________

Thorax:

Inspection (abnormal/extra nipples, hair, etc) □Yes □No _______________________________

St.c: □i.a. □Abnormal _______________________________

St.p: □i.a. □Abnormal _______________________________

Pectus excavatum □Yes □No _______________________________

Pectus carinatum □Yes □No _______________________________

Other □Yes □No _______________________________

Abdomen:

Omphalocele □Yes □No _______________________________

Other □Yes □No _______________________________

Gonad:

Hypogonadism □Yes □No _______________________________

Other □Yes □No _______________________________

Skin and lymph-nodes:

Telangiectasia □Yes □No _______________________________

Café au lait spots □Yes □No _______________________________

Hypopigmentation □Yes □No _______________________________

Freckles (axillary, inguinale) □Yes □No _______________________________

Nail-dystrophy □Yes □No _______________________________

Hair color □ White □ Blond □ Red □ Brown □ Black

Hair quality □ Fine □ regular □ Coarse

Eczema □Yes □No _______________________________

Hyperpigmentation □Yes □No _______________________________

Tumors/papules of the skin □Yes □No _______________________________

Other □Yes □No _______________________________

Extremities:

Hand abnormalities □Yes □No _______________________________

Foot abnormalities □Yes □No _______________________________

Bone dysplasia □Yes □No _______________________________

No/abnormal radial ray □Yes □No _______________________________

Hypotonia □Yes □No _______________________________

Dystonia □Yes □No _______________________________

Four finger furrows □Yes □No _______________________________

Other □Yes □No _______________________________

Neurology:

Ataxia □Yes □No _______________________________

Seizures □Yes □No _______________________________

Other □Yes □No _______________________________

Other relevant findings (not covered by the checklist):
