# Supplementary material for: Nationwide germline whole genome sequencing of 198 consecutive pediatric cancer patients reveals a high incidence of cancer prone syndromes
Source: PLoS Genet. 2020 Dec 17;16(12):e1009231. doi: 10.1371/journal.pgen.1009231 (PMC7787686; doi:10.1371/journal.pgen.1009231)
Supplement: Attachment — Submitted filename: review notes.docx [file pgen.1009231.s003.docx]

Byrjalsen, A. et al. performed whole-genome sequencing (WGS) of germline DNA on first 198 newly diagnosed pediatric cancer patients included in the Danish, prospective, nationwide study "Sequencing Tumor and Germline DNA-Implications for National Guidelines (STAGING)." This study was the first nationwide unselected cohort of consecutive pediatric cancer patients in Denmark based on WGS, clinical examination, and pedigree mapping. The authors did a great job comprehensively analyzing the germline variants of these patients. They showed that about half of those patients carried pathogenic variants in cancer predisposition syndromes (CPS) genes or had clinical features indicating CPS. They also showed 14.6% of those patients harbored a CPS. This study indicates that a higher proportion of childhood cancers may be linked to predisposing germline variants than previously supposed.

Specific points:

1. In Discussion, the authors mentioned that "The frequency of PVs in our study was significantly higher than that observed in control cohorts wherein 0.6–1.1% of adult patients in a Genomics England cohort and a cohort of pediatric and adult patients with autism had PVs in CPS genes". These are not valid comparisons, as the methods and analysis approaches were very different in these studies, and it needs to be clarified in the text. Later in the Discussion, the authors talked about the limitation of their study "the lack of a comparison cohort, because population-based WGS data from ethnically comparable children are not publicly available," but it needs to be addressed in the above paragraph as well.

1. This is a confusing sentence and needs to be clarified and re-written: "A high frequency of PVs in BRCA2 in the general population41 would not explain why many studies have identified so few PVs in BRCA1, which are more frequently associated with breast cancer than BRCA2 PVs in the Danish population".

1. Criteria of analysis with VarSeq has not been explained well in the Methods and Materials (other than CADD and allele frequency). Please include other criteria, if there were any.

1. ClinVar could have been used for further filtration. For example, some of the VUS included in the supplementary materials have benign or likely benign classification based on the Clinvar and could have been eliminated from the results.

1. There are two mentioned *P* values in the study, but no further details about the MM section's statistical method.

Minor points:

1. Figure 2 does not have publication quality and needs to be modified for the publication.
2. Table 2 does not have a Table format, and it needs to be modified for the publication
3. Table 8 needs a column with patients' number or code
