# Supplementary material for: Nationwide germline whole genome sequencing of 198 consecutive pediatric cancer patients reveals a high incidence of cancer prone syndromes
Source: PLoS Genet. 2020 Dec 17;16(12):e1009231. doi: 10.1371/journal.pgen.1009231 (PMC7787686; doi:10.1371/journal.pgen.1009231)
Supplement: Attachment — Submitted filename: Response to reviewer comments.docx [file pgen.1009231.s004.docx]

Aries Systems Corporation


Coronavirus (COVID-19) Update from Aries Systems

# Aries Systems Corporation

- Request a Demo
- Contact Us

Toggle navigation

- Solutions
  - Solutions
  - Editorial Manager
  - LiXuid Manuscript
  - ProduXion Manager
  - Task Manager
  - Commerce Manager
  - Standards
- Journals List
- Support & Resources
  - Client Services
  - Resources
  - User Community
- Videos
- Views & Press
  - News & Opinion
  - Press Releases
  - Newsletter Archive
- Events
- About
  - About Aries
  - Partners
  - Testimonials
  - Privacy Policy
  - Careers

Search All 

News & Opinion 

Press Releases 

FAQs 

Pages 

Posts 

Files 

Videos 

Newsletter

Search
Clear

- Home
- Solutions
  - Solutions
  - Editorial Manager
  - LiXuid Manuscript
  - ProduXion Manager
  - Task Manager
  - Commerce Manager
  - Standards
  - User Community
- Journals List
- Support & Resources
  - Client Services
  - Resources
  - Videos
  - User Community
- Videos
- Views & Press
  - News & Opinion
  - Press Releases
  - Newsletter Archive
- Events
- About
  - About Aries
  - Partners
  - Testimonials
  - Privacy Policy
  - Careers
- Request a Demo
- Contact Us
- Careers
- Search All 

  News & Opinion 

  Press Releases 

  FAQs 

  Pages 

  Posts 

  Files 

  Videos 

  Newsletter

  Search
  Clear

# Publish Faster. Publish Smarter.

## Workflow infrastructure solutions for scholarly publishers

Manuscript Submission   
& Peer Review

Comprehensive XML   
Workflow Solution

Manuscript Production   
& Task Tracking

View All Solutions

## Videos

Browse hundreds of videos showcasing Editorial Manager and ProduXion Manager system features and functionality

Watch Videos

## Journals List

Looking for a specific journal using Editorial Manager? Search our Journals List to locate a publication’s EM/PM portal

See Journals List

## Testimonials

Don’t take our word for it. See what current users are saying about their experience with Editorial Manager and ProduXion Manager

Read Reviews

## FAQs

Have a question? Find answers to our most common queries about access and support, GDPR, adopting a new system and more!

View FAQs

## You’re in Good Company

## Recent News & Press

## Preventing Manuscript Redundancy in EM with Duplicate Submission Check

## The Benefits of an XML-First Publishing Workflow

## Meet the Aries Team: Craig Mack, Senior Database Administrator

## EEMUG 2021 Webinar Series: Registration Now Open!

View All News & Opinion


## Looking for More Information?

We’re happy to answer questions and provide free demonstrations.

Request a Demo

- Privacy Policy
- Japanese Version

- Aries Systems Corporation
- 50 High Street, Suite 21
- North Andover, MA, 01845

© 2020 Aries Systems Corporation. Editorial Manager, LiXuid Manuscript, ProduXion Manager, and Commerce Manager are trademarks and the property of Aries Systems Corporation. All Rights Reserved. Subject to change without notice from or liability to Aries Systems Corporation.
